# Ginsenoside Rh4 Suppresses Metastasis of Gastric Cancer via SIX1-Dependent TGF-β/Smad2/3 Signaling Pathway

**DOI:** 10.3390/nu14081564

**Published:** 2022-04-09

**Authors:** Hongbo Jiang, Pei Ma, Zhiguang Duan, Yannan Liu, Shihong Shen, Yu Mi, Daidi Fan

**Affiliations:** 1 Shaanxi Key Laboratory of Degradable Biomedical Materials, Shaanxi R&D Center of Biomaterials, Fermentation Engineering, School of Chemical Engineering, Northwest University, Taibai North Road 229, Xi’an 710069, China; jianghongbo@stumail.nwu.edu.cn (H.J.); mapei@nwu.edu.cn (P.M.); duanzhiguang@nwu.edu.cn (Z.D.); liuyannan@nwu.edu.cn (Y.L.); shenshihong@nwu.edu.cn (S.S.); 2 Biotech and Biomed Research Institute, Northwest University, Taibai North Road 229, Xi’an 710069, China

**Keywords:** ginsenoside Rh4, SIX1, TGF-β/Smad2/3 pathway, gastric cancer

## Abstract

Gastric cancer (GC) is the leading causes of cancer-related death worldwide. Surgery remains the cornerstone of gastric cancer treatment, and new strategies with adjuvant chemotherapy are currently gaining more and more acceptance. Ginsenoside Rh4 has excellent antitumor activity. Conversely, the mechanisms involved in treatment of GC are not completely understood. In this study, we certified that Rh4 showed strong anti-GC efficiency in vitro and in vivo. MTT and colony formation assays were performed to exhibit that Rh4 significantly inhibited cellular proliferation and colony formation. Results from the wound healing assay, transwell assays, and Western blotting indicated that Rh4 restrained GC cell migration and invasion by reversing epithelial–mesenchymal transition (EMT). Further validation by proteomic screening, co-treatment with disitertide, and SIX1 signal silencing revealed that SIX1, a target of Rh4, induced EMT by activating the TGF-β/Smad2/3 signaling pathway. In summary, our discoveries demonstrated the essential basis of the anti-GC metastatic effects of Rh4 via suppressing the SIX1–TGF-β/Smad2/3 signaling axis, which delivers a new idea for the clinical treatment of GC.

## 1. Introduction

Gastric cancer (GC) remains a non-negligible cancer worldwide, ranking fifth for incidence and fourth for mortality globally. As reported by *CA: A Cancer Journal for Clinicians*, there were over one million new cases in 2020 with an estimated 769,000 deaths caused by GC [1]. Despite the great advancement in the treatment and diagnosis of GC in recently years, the morbidity and mortality of GC have remained uncontrolled. Metastasis and resistance to chemotherapeutic agents are major problems in GC treatment [2]. Therefore, it is necessary to deeply understand the mechanisms of GC metastasis and identify therapeutic agents to achieve precise treatment of patients and prolong their survival time.

Epithelial–mesenchymal transition (EMT) plays a key role in cellular processes associated with cancer metastasis and progression, in which tumor cells lose epithelial features, accompanied by reduced cell junctions and acquisition of invasive and metastatic capabilities, as well as stem-cell phenotypes [3,4]. GC genesis, progression, and metastasis are closely related to EMT. The levels of transforming growth factor-β1 (TGF-β1), Snail1 (Snail, a key regulator in EMT), and Vimentin (an important component protein of cytoskeleton) are all upregulated in patients with dysplasia or early GC, while the level of E-cadherin is decreased [5]. When aberrant EMT is activated, cadherin switches from E-cadherin to N-cadherin, which increases cell motility and invasiveness in GC progression [6]. Zhang et al. demonstrated that CCR7 upregulates Snail expression to induce EMT, leading to GC progression, migration, and invasion [7]. Accumulating evidence has confirmed TGF-β1 to be a key growth factor which induces EMT of various tumors [8,9,10]. It has been reported that TGF-β1-induced EMT is dependent on the downstream effector, Smad. TGF-β receptor II (TGF-βRII) phosphorylates TGF-β receptor I (TGF-βRI) in response to TGF-β1, which in turn activates Smads. These activated receptor-regulated Smads (R-Smads) form complexes that then act as transcriptional regulators ectopically into the nucleus to regulate the expression of EMT-related proteins such as epithelial cadherin (E-cadherin) and Snail [11,12]. Furthermore, a study by Irina et al. revealed that the TGF-β1-induced cell invasion and EMT phenotype in lung cancer cells are Smad2/3-dependent [13].

Sineoculis homeobox homolog 1 (SIX1) is a member of the *SIX* gene superfamily located on human chromosome 14 [14], and it is accepted as a transcription factor involved in the regulation of embryonic development [15]. Among the recently studied molecules reportedly linked to EMT is SIX1 [16]. As recorded in multiple studies, SIX1 can be highly expressed in different tumors, including liver cancer, colorectal cancer, gastric cancer, and breast cancer, thus affecting their clinical prognosis [14,17] and signal transduction [18], as well as promoting tumor progression. Furthermore, SIX1-induced upregulation of TGF-β1 is critical for propagation of TGF-β/Smad2/3 signaling, induction of EMT persistence, and metastasis [19,20]. Farabaugh et al. showed that SIX1 induces EMT by activating the TGF-β signaling pathway and interacting with its pro-metastatic function, thereby causing cancerous epithelial cells to become cancerous mesenchymal cells [20,21]. Although some studies in other cancers indicated that SIX1 regulates EMT processes through the TGF-β/Smad2/3 signaling pathway in papillary thyroid cancer [22], there is no direct evidence that SIX1 induces EMT via the TGF-β/Smad2/3 signaling pathway in GC. Thus, it is necessary to investigate the effects of SIX1 and TGF-β/Smad2/3 on EMT in GC.

Ginseng is a medicinal plant widely used in traditional and modern medicine, and its active ingredients are its secondary metabolites, ginsenosides, which have excellent pharmacological effects in clinical applications, such as for the treatment of cardiovascular diseases [23], as well as antitumor [24,25], antidiabetic [26], anti-inflammatory [27,28,29], anti-obesity [30,31], and neuroprotective effects [32], and immunity enhancement [33]. Ginsenoside Rh4 is known to be a tetracyclic triterpenoid saponin composed of a glucosin and a triterpene sapogenin [34]. Earlier studies showed that ginsenosides affect EMT through inhibiting TGF-β1 expression [35], activating PI3K/Akt [36], and downregulating NF-κB [37]. However, there are no reports about the anti-metastasis effect of Rh4 on GC, and its mechanism remains unknown. In our study, the results showed that Rh4 could target SIX1 to inhibit the TGF-β/Smad2/3 signaling pathway and effectively suppressed EMT metastasis in GC cells according to the wound healing assay, transwell assay, proteome sequencing, and Western blot.

## 2. Materials and Methods

### 2.1. Materials and Chemicals

Ginsenoside Rh4 (Supplementary Figure S1A, purity ≥ 99%) was purchased from Puruifa Technology Development Co., Ltd. (Chengdu, China). Methylthiazoletetrazolium (MTT) and dimethyl sulfoxide (DMSO) were obtained from Aladdin Biotechnology (Shanghai, China). Fetal bovine serum (FBS), streptomycin, and penicillin were purchased from Gibco (Grand Island, NY, USA). Dulbecco’s modified Eagle medium (DMEM) was purchased from HyClone (Logan, UT, USA). The 24-well Transwell chambers were obtained from Corning (New York, NY, USA). Lipofectamine 2000 and TRIzol Reagent were supplied by Invitrogen (Carlsbad, CA, USA). SYBR Green Master Mixture was obtained from Takara (Otsu, Japan). Primary antibodies against E-cadherin, Vimentin, N-cadherin, Snail1, SIX1, and TGF-β were purchased from Proteintech Group Inc. (Chicago, IL, USA). The primary antibodies against Smad3, P-Smad3, and β-actin were supplied by Abcam (Cambridge UK). Goat anti-mouse IgG and goat anti-rabbit IgG were obtained from Abbkine (Wuhan, China). Oxaliplatin was obtained from Qilu Pharmaceutical Co., Ltd. (Shandong, China).

### 2.2. Cell Culture

Human gastric carcinoma cell lines (HGC-27, BGC-823) were supplied by the American Type Culture Collection (ATCC, VA, USA). Cells were cultured in DMEM with 10% FBS and 1% penicillin/streptomycin and maintained in an atmosphere of 5% CO_2_ at 37 °C.

### 2.3. Cell Proliferation Assay

HGC-27 and BGC-823 (1 × 10^5^ cells/well) were seeded in 96-well plates. After adherence, different concentrations of Rh4 (0, 20, 40, 60, 80, 100, and 120 µM) were injected into each well for 24 h. Then, 50 µL of MTT (5 mg/mL) and 150 µL of DMSO were added into the plates sequentially. Absorbance was measured at 490 nm using a microplate reader (Power Wave XS2, Bio-Tek Instruments Inc., Winooski, VT, USA).

### 2.4. Colony Formation Assay

HGC-27 and BGC-823 (500 cells/well) were seeded evenly in six-well plates and incubated overnight. Then, cells were cocultured with Rh4 (0, 40, 80, and 120 µM) for 48 h. Finally, cells were fixed, stained, and counted when visible colonies formed. The independent experiment was repeated three times.

### 2.5. AO/EB Staining

HGC-27 and BGC-823 (2 × 10^5^ cells/well) were cultured evenly in six-well plates and incubated overnight. After adherence, cells treated with Rh4 (0, 40, 80, and 120 µM) for 24 h. Then, each well was added with an acridine orange/ethidium bromide (AO/EB) mixture for 30 to 45 min, before photographing the results. Three separate experiments were conducted.

### 2.6. Wound Healing Assay

GC cell lines (HGC-27, BGC-823) clones were grown to confluency. A linear wound was made by scraping a non-opening Pasteur pipette across the confluent cell layer. Cells were washed twice to remove detached cells and debris, treated with serum-free DMEM medium containing different concentrations of Rh4 (0, 20 μM, and 40 μM). Then, the sizes of the wounds at the same location were observed and measured at the indicated times (0 h, 12 h, 24 h) by ImageJ software. The independent experiment was repeated three times.

### 2.7. Transwell Assay

HGC-27 and BGC-823 (5 × 10^3^/2 × 10^4^ cells per well) were seeded without or with Matrigel coating (1 mg/mL, BD Matrigel ™) in the top chamber of the Transwell. Then, 600 μL of DMEM with 10% FBS was added to the Transwell lower chamber. The migrates/invasive cells were fixed, stained, and counted by an inverted microscope (200× magnification), 24 h later. At least three independent experiments were conducted [38,39].

### 2.8. Western Blot

The total protein of HGC-27 and BGC-823 was collected and extracted using lysis buffer. Cell extracts were measured by the BCA kit (Beyotime, Shanghai, China), and equal quantities of proteins (15 μg) were separated by 10% SDS-PAGE. The isolated proteins were transferred onto PVDF membranes by Trans-Blot Turbo (BioRad, Hercules, CA, USA). PVDF membranes were sequentially incubated with different primary antibodies, eluted with buffer, incubated with HRP-conjugated secondary antibodies, eluted with buffer, and imaged by a Gel Image system (Tanon5200, Shanghai, China). Data were expressed as the mean ± SD of at least three independent experiments.

### 2.9. Quantitative Real-Time Polymerase Chain Reaction (qRT-PCR)

Total RNA was derived from HGC-27 and BGC-823, followed by reverse transcription to obtain cDNA. The relative mRNA levels were calculated using the 2^−ΔΔCT^ method. The expression of β-actin was used as an internal control. β-actin: 5′–CTGTCCCTGTATGCCTCT–3′ (forward), 5′–ATGTCACGCACGATTTCC–3′ (reverse); E-cadherin: 5′–GGAACTATGAAAAGTGGGCTTG–3′ (forward), 5′–GGCATCAGGCTCCACAGT–3′ (reverse); N-cadherin: 5′–GGTGGAGGAGAAGAAGACCAG–3′ (forward), 5′–GGCATCAGGCTCCACAGT–3′ (reverse); Vimentin: 5′–GACGCCATCAACACCGAGTT–3′ (forward), 5′–CTTTGTCGTTGGTTAGCTGGT–3′ (reverse); Snail1: 5′–CTTCCAGCAGCCCTACGAC–3′ (forward); 5′–CGGTGGGGTTGAGGATCT–3′ (reverse). Detail of qRT-PCR method was described in the supplementary method 1.1.

### 2.10. Proteomics Analysis

Cell lysis and digestion: The HGC-27 cellular pellet was resuspended in boiling lysis buffer and boiled for 10 min before further lysis by sonication with a microtip probe. The protein concentration was determined using a BCA kit.

iTRAQ labeling of peptides and strong cation exchange (SCX) chromatography: Aliquots of 100 µg of peptide from each grade were labeled using the iTRAQ assay. The labeled peptides were incubated for 2 h at room temperature, then mixed in equal proportions, and dried by vacuum centrifugation. Further separation of iTRAQ-labeled peptides was achieved using high performance liquid chromatography.

LC–MS/MS analysis: All samples were evaluated on an Easy Nano Liquid Chromatography apparatus (nLC) coupled to a Q-Exactive HF-X MS (Thermo Fisher Scientific, Waltham, MA, USA) 20.

Data processing and bioinformatics analysis of proteins: The mass spectra were analyzed with MaxQuant software version 1.5.8.3 (Max Planck Institute of Biochemistry, Munich, Bavaria, Germany). Bioinformatics analysis was performed using the Gene Ontology (GO) database to annotate the differentially expressed proteins. Unsupervised hierarchical clustering analysis, PCA, and volcano plot analysis were performed in Perseus. The Kyoto Encyclopedia of Genes and Genomes (KEGG) database provided the analysis of the pathways of differentially expressed proteins. Enrichment analyses for GO and KEGG pathways were carried out to reveal the enriched pathways of significantly altered proteins and to identify their functions. Detail of proteomics anailsis was decribed in the supplementary method 1.2.

### 2.11. SiRNA Transfection

The specific siRNA of SIX1 (sense: 5′–GUCAGCAACUGGUUUAAGATT–3′ and antisense: 5′–UCUUAAACCAGUUGCUGACTT–3′) was provided by GenePharma (Shanghai, China). HGC-27 and BGC-823 were transfected for 48 h with Lipofectamine ™ 2000 (Carlsbad, CA, USA) and 100 pM siRNA duplexes in six-well plates, and then treated with or with Rh4 (40 μM) for follow-up research.

### 2.12. Tail Vein Injection Model

This experiment was conducted with permission from the Animal Ethics Committee of Northwest University (NWU-AWC-20210310M) and complied with the requirements of the Law of the People’s Republic of China on Laboratory Animals. Male BALB/c nude mice (5 weeks old, weighing about 21 g) were acquired from GemPharmatech (Jiangsu, China). Lung metastasis was studied by washing 2 × 10^5^ HGC-27 in serum-free DMEM and injecting it intravenously into mice (*n* = 40). The successfully modeled mice were randomly divided into the following six groups (*n* = 10, intraperitoneal injection): normal group, normal + Rh4 group (100 mg per kg daily), control group (model mice), low-dose Rh4 group (50 mg per kg daily), high-dose Rh4 group (100 mg per kg daily), and cisplatin group (10 mg per kg per 3 days) [40]. All mice were euthanized by continuous injection for 3 weeks, and critical organs were isolated and weighed. Then, some organs were paraffin-embedded, while some were stored at −80 °C. Bouin’s solution was used to fix and stain some lungs, whose metastases were distinguished as white colonies.

### 2.13. Histopathology and Immunohistochemistry Assay (IHC)

The major organs (heart, lung, spleen, kidney, and liver) of nude mice were isolated, fixed in 4% paraformaldehyde, weighed, and paraffin-embedded. For H&E, sectioning and staining were applied. For IHC, paraffin was sectioned, pretreated, incubated with primary antibody, incubated with secondary antibody, stained, and photographed under an optical microscope (Nikon, Japan).

### 2.14. Immunofluorescence Assay (IF)

HGC-27 and BGC-823 were adhered to the slides and cocultured with different concentrations of Rh4 for 24 h. Subsequently, cells were fixed, blocked in 5% bovine serum albumin, incubated with primary antibody, incubated with secondary antibody, and photographed for analysis by Olympus confocal microscopy (Tokyo, Japan).

### 2.15. Statistical Analysis

Statistical analysis was performed by SPSS 20.0 (Chicago, IL, USA) and Prism 9 (San Diego, CA, USA) software. Differences between groups were derived from one-way analysis of variance (ANOVA). Results are from at least three separate experiments and are expressed as the mean ± standard deviation (SD). A *p*-value <0.05 reveals statistically significant differences.

## 3. Results

### 3.1. Ginsenoside Rh4 Inhibited GC Cell Growth In Vitro

To investigate the antiproliferative activity of Rh4 on GC cells, HGC-27 and BGC-823 were incubated with different concentrations of Rh4 for 24 h and subjected to MTT assay. The results demonstrated that Rh4 significantly reduced the vigor of HGC-27 and BGC-823 in a dose- and time-dependent manner (Supplementary Figure S1B,C). The IC_50_ values for Rh4-treated HGC-27 and BGC-823 were 83.15 μM and 92.38 μM, respectively. Moreover, compared to the control, the analysis of colony formation assay revealed that Rh4 suppressed the formation of HGC-27 and BGC-823 colonies (Supplementary Figure S1D). Meanwhile, AO/EB staining experiments proved that Rh4 significantly reduced the viability of HGC-27 and BGC-823 in a dose-dependent manner (Supplementary Figure S1E). In brief, these results confirmed that Rh4 has a strong antitumor effect on gastric cells in vitro.

### 3.2. Ginsenoside Rh4 Restrains Migration and Invasion of GC Cells

**Figure 1:**
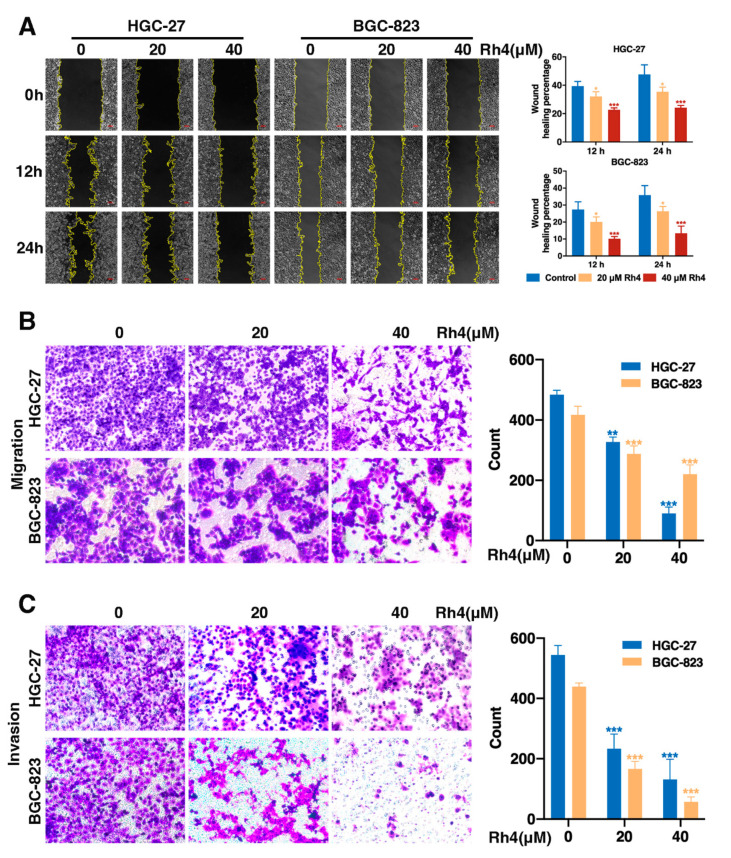
Ginsenoside Rh4 inhibits GC metastasis in vitro. Wound healing assays (**A**) and transwell migration (**B**) and invasion (**C**) assays were performed to investigate the migration and invasion ability alteration of BC cells with the treatment of Rh4. Data were processed using ImageJ software (NH, Bethesda, MD, USA). Quantification charts are listed on the right. Statistics are exhibited as the mean ± SD of triplicate independent experiments; * *p* < 0.05, ** *p* < 0.01, *** *p* < 0.001.

To test the antimigration and anti-invasion functions of Rh4 on GC cells, wound healing assays and transwell assays were conducted. HGC-27 and BGC-823 were incubated with Rh4 at concentrations of 20 and 40 μM, respectively. The wound healing assay showed that the wound healing percentages decreased evidently in a dose-dependent manner (Figure 1A, * *p* < 0.05, *** *p* < 0.001). Furthermore, the transwell assay for migration proved that the cell metastasis ability of the Rh4 treatment group showed a dose-dependent reduction of 32.44–81.4% in HGC-27 and 30.97–47.24% in BGC-823 (Figure 1B, ** *p* < 0.05, *** *p* < 0.001), indicating the inhibitory effect of Rh4 on migration. A Matrigel invasion assay was carried out to evaluate the ability of HGC-27 and BGC-823 to invade through the Matrigel and membrane barrier of the transwell. The results revealed that the treatment of Rh4 could effectively attenuate HGC-27 and BGC-823 invasion in a dose-dependent manner (Figure 1C, *** *p* < 0.001). These results demonstrated that the migration and invasion ability were limited in a dose-dependent manner after treatment with Rh4 in HGC-27 and BGC-823.

### 3.3. Ginsenoside Rh4 Inhibited GC Metastasis In Vivo, and Causes Low Toxicity and Side-Effects

A tail vein injection model was built to verify the impact of ginsenoside Rh4 on GC tumor metastasis in vivo [41]. Compared to the control, the number of lung nodules treated with Rh4 and oxaliplatin (drug commonly used in adjuvant therapy for gastric cancer) showed an obvious reduction (Supplementary Figure S2A,B). These results suggest that Rh4 suppressed tumor growth and metastasis in vivo.

Furthermore, the body weight of mice treated with low and high doses of Rh4 were not significantly different from the control, while the oxaliplatin group showed a dramatic decrease, indicating no systemic toxicity after Rh4 administration (Supplementary Figure S2C). The H&E staining of organ tissues showed that the heart, spleen, and lung damage were visible in the oxaliplatin group, compared with no effect in the Rh4 group (Supplementary Figure S2D). Additionally, there were no statistically significant differences in organ indicators between each group, except for a significantly lower splenic index in the oxaliplatin group (Table 1). In summary, these results demonstrated that Rh4 causes no toxic side-effects in GC treatment.

### 3.4. Ginsenoside Rh4 Reverses EMT In Vitro and In Vivo

**Figure 2:**
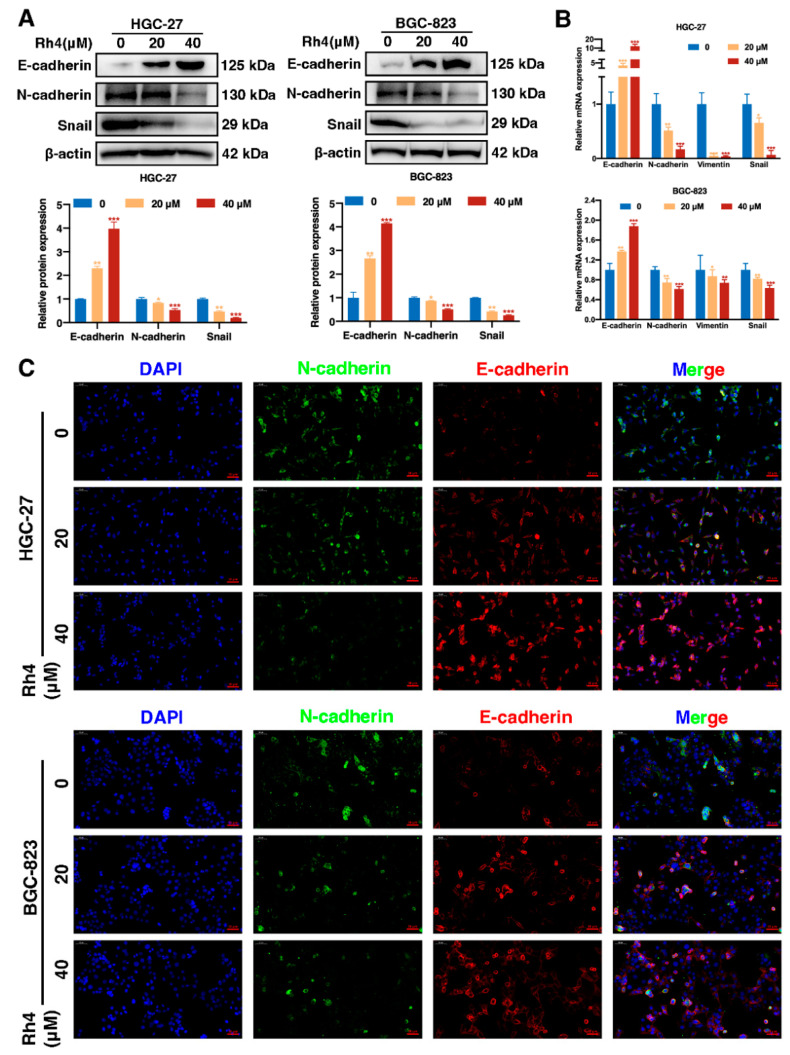
Ginsenoside Rh4 reverses EMT in vitro. (**A**) Western blot. Quantification plots are shown below. (**B**) qRT-PCR assay. β-actin was used as an endogenous reference. (**C**) IF assay. E-cadherin (red) and N-cadherin (green) are presented. DAPI (blue) was used to mark the cell nuclei. Scale bars = 50 µm. Statistics are exhibited as the mean ± SD of triplicate independent experiments; * *p* < 0.05, ** *p* < 0.01, *** *p* < 0.001.

To evaluate whether ginsenoside Rh4 inhibits EMT in HGC-27 and BGC-823, the expression of EMT-related proteins and mRNA was studied. Rh4 attenuated the expression of N-cadherin and Snail and enhanced that of E-cadherin (epithelial marker) in a dose-dependent manner in HGC-27 and BGC-823 (Figure 2A). Additionally, qRT-PCR analysis showed that Rh4 treatment (20 and 40 µM) upregulated the transcription level of E-cadherin and downregulated the transcription levels of N-cadherin, Vimentin, and Snail in HGC-27 and BGC-823 (Figure 2B). Moreover, changes in the protein content of HGC-27 and BGC-823 were more clearly assessed by immunofluorescence (IF), and it was observed that Rh4 decreased E-cadherin levels and increased N-cadherin levels (Figure 2C). Overall, it was demonstrated that ginsenoside Rh4 specifically interfered with the expression of N-cadherin, E-cadherin, and other biomarkers to reverse the EMT process.

**Figure 3:**
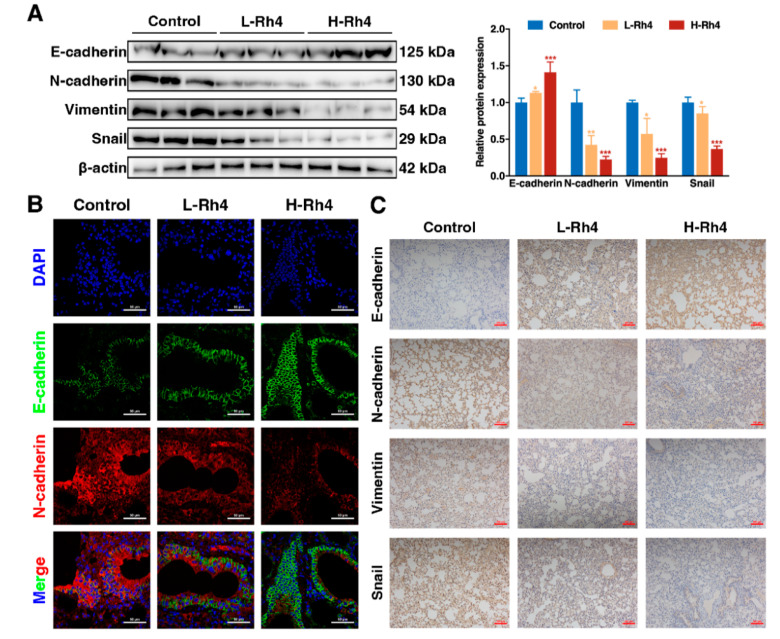
Ginsenoside Rh4 reverses the EMT procedure of GC in vivo. (**A**) Western blot showing increased E-cadherin and decreased N-cadherin, Vimentin, and Snail in tumor tissues under Rh4 treatment. β-Actin was used as an endogenous reference. Quantification plots are shown on the right. (**B**) IF assay. The downward adjustment of E-cadherin and upward adjustment of N-cadherin were reversed by Rh4 in vivo. Scale bars = 50 µm. (**C**) IHC assays were performed to assess changes in E-cadherin, N-cadherin, Vimentin, and Snail. Scale bars = 200 µm. Statistics are exhibited as the mean ± SD of triplicate independent experiments; * *p* < 0.05, ** *p* < 0.01, *** *p* < 0.001.

Lung tissue samples of the HGC-27 tail vein injection model were analyzed by Western blot (Figure 3A), IF (Figure 3B), and immunohistochemical analysis (Figure 3C). The results showed consistent trends in EMT-related proteins in vivo and in vitro.

### 3.5. SIX1 Is Involved in EMT Suppression by Rh4

**Figure 4:**
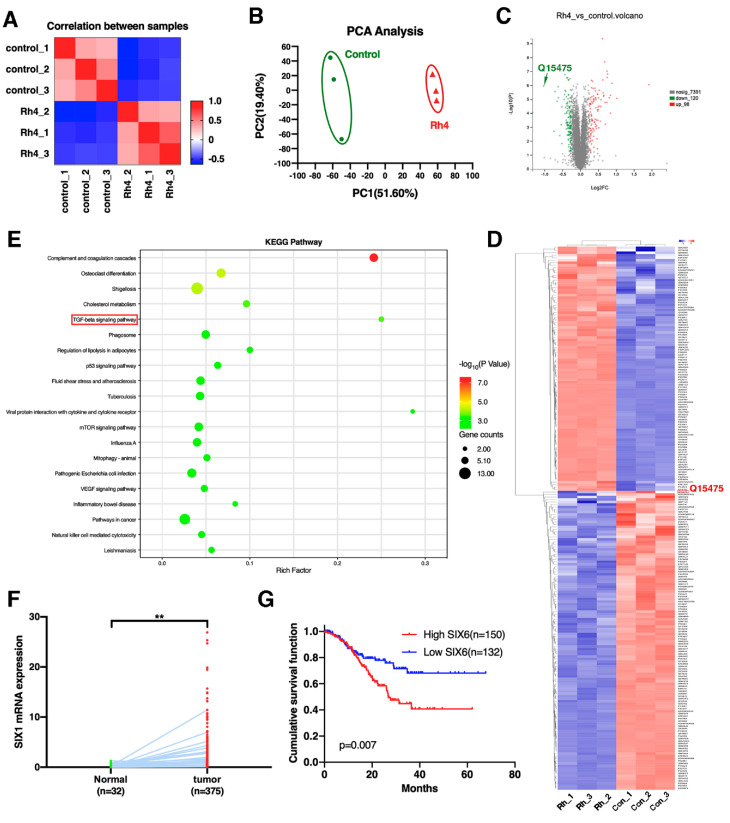
Proteomics and clinical data indicating that ginsenoside Rh4 targeted SIX1, suppressing EMT in GC. (**A**) Correlation heatmap of samples. (**B**) Principal component analysis (PCA) of significantly regulated proteins in the HGC-27 groups with or without Rh4 treatment (*n* = 3 biologically independent experiments). (**C**)Volcano plot analysis for the Rh4 treatment/control comparisons. Proteins regulated on the proteome level are marked in red (upregulated) and green (downregulated). Q15475 represents the SIX1 protein. (**D**) Heatmap hierarchical clustering from differentially expressed proteins (*n* = 218) identified across all sample groups. Q15475 represents the SIX1 protein. (**E**) Statistics of KEGG pathway enrichment. (**F**) TCGA analysis of SIX1 expression levels in GC tissues and paracancerous tissues (** *p* < 0.01). (**G**) Survival curves synthesized using Kaplan–Meier method.

To further investigate how Rh4 inhibits EMT in GC cells, proteomic analysis was performed using quantitative mass spectrometry (MS) of the proteome (Figure 4A). Three biological replicates were collected per condition. In the dataset, 7609 proteins were quantified. In order to assess whether the response to Rh4 is obvious at the proteomic level, principal component analysis (PCA) was performed by the sample (Figure 4B). This analysis showed that the different sample proteomes were separated into two distinct groups on component 1, reflecting their response to Rh4. Next, differentially regulated proteins were identified between the control and Rh4-treated cell groups by performing a microarray significance analysis (SAM) statistical test. A total of 218 regulated proteins were found, with 98 in the Rh4-treated cell group. Significantly more proteins (120) were downregulated (Figure 4C). To determine how Rh4 inhibits GC metastasis, enrichment analysis of the KEGG signaling pathway was performed (Figure 4E). It was found that the TGF-β signaling pathway was the most significantly enriched. At the same time, the upstream protein SIX1 of the TGF-β signaling pathway was regulated by Rh4 (Figure 4C,D). TCGA dataset showed that SIX1 expression levels in normal tissues were higher than in tumor tissues (*p* = 0.0018) (Figure 4F). Kaplan–Meier survival analysis showed that patients with high SIX1 expression had a shorter overall survival period (Figure 4G). These results suggest that ginsenoside Rh4 may suppress EMT of GC cells via mediating the TGF-β/Smad2/3 signaling pathway by targeting SIX1.

### 3.6. Ginsenoside Rh4 Reverses EMT through SIX1–TGF-β/Smad2/3 Signaling Axis In Vitro and In Vivo

**Figure 5:**
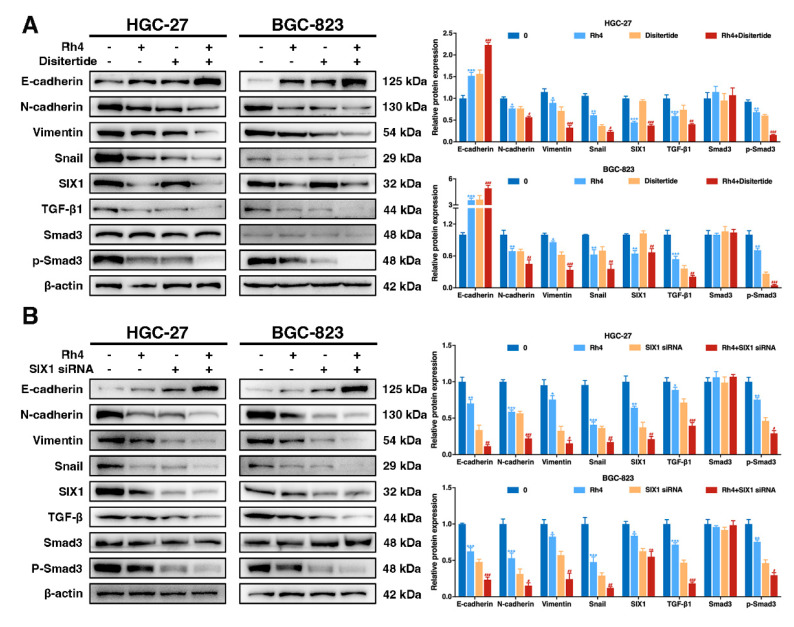
SIX1 is critical for Rh4-induced TGF-β/Smad2/3 signaling pathway and EMT process inhibition in GC. (**A**) Western blot showing the effects of EMT biomarkers and TGF-β/Smad2/3 signaling protein expression in GC cells (HGC-27 and BGC-823) after incubation with disitertide and Rh4. (**B**) Western blot showing effects of EMT biomarkers and TGF-β/Smad2/3 signaling protein expression in GC cells (HGC-27 and BGC-823) after incubation with SIX1-siRNA and Rh4. β-Actin was used as an endogenous reference. Quantification plots are shown on the right. Statistics are exhibited as the mean ± SD of triplicate independent experiments; * *p* < 0.05, ** *p* < 0.01, and *** *p* < 0.001 compared with the control group; # *p* < 0.05, ## *p* < 0.01, and ### *p* < 0.001 compared with the disitertide-treated group.

To verify the mechanism of action of Rh4 and whether SIX1 is a key target for regulation and antitumor activity, the related protein expression under the combined treatment of Rh4 and TGF-β1 inhibitor (disitertide) was evaluated. Expression of TGF-β1 and P-Smad3 was restrained in the disitertide group compared to the disitertide-negative control, and levels of these TGF-β/Smad2/3 pathway-related proteins were further downregulated by Rh4 (40 μM). Complementarily, coincubation with disitertide and Rh4 enhanced the effect of Rh4 treatment alone on E-cadherin, N-cadherin, Vimentin, and Snail. (Figure 5A). The disitertide group had no effect on the expression of SIX1, while the siRNA transfection assay of SIX1 displayed that Rh4 restrained SIX1 expression specifically (Figure 5B), proving that SIX1 is the target protein of Rh4. The outcomes demonstrated that ginsenoside Rh4 reverses the EMT of GC cells via regulating the TGF-β/Smad2/3 signaling pathway by targeting SIX1.

**Figure 6:**
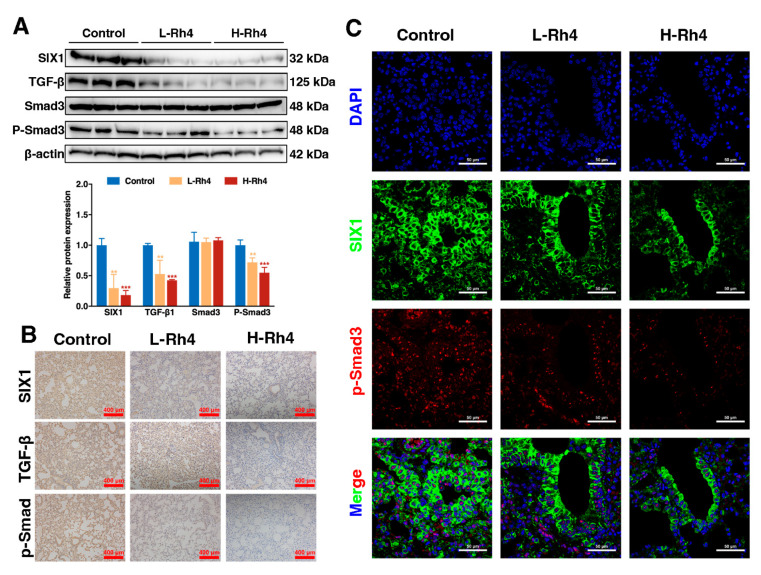
Ginsenoside Rh4 attenuated TGF-β/Smad2/3 signaling pathway of GC in vivo. (**A**) Western blot showing expression of SIX1, TGF-β, Smad3, and P-Smad3 in tumor tissues under Rh4 treatment. (**B**) IHC assay performed to assess changes in SIX1, TGF-β, and P-Smad3. Scale bars = 200 µm. (**C**) IF assay. Upregulation of SIX1 and P-Smad3 is reversed by Rh4 in vivo. Scale bars = 50 µm. Statistics are exhibited as the mean ± SD of triplicate independent experiments; ** *p* < 0.01, *** *p* < 0.001.

To further assess whether Rh4 inhibited the metastasis of GC cells in vivo, lung tissue samples of the HGC-27 tail vein injection model were analyzed by Western blot, IF, and IHC assays. The lung tissue treated with Rh4 showed weak TGF-β/Smad2/3 signaling pathway signals, as evidenced by Western blot (Figure 6A), IHC (Figure 6B), and IF assays (Figure 6C), consistent with the results in vitro. This means that the metastasis of GC cells in vivo can be regulated by treatment with Rh4.

## 4. Discussion

**Figure 7:**
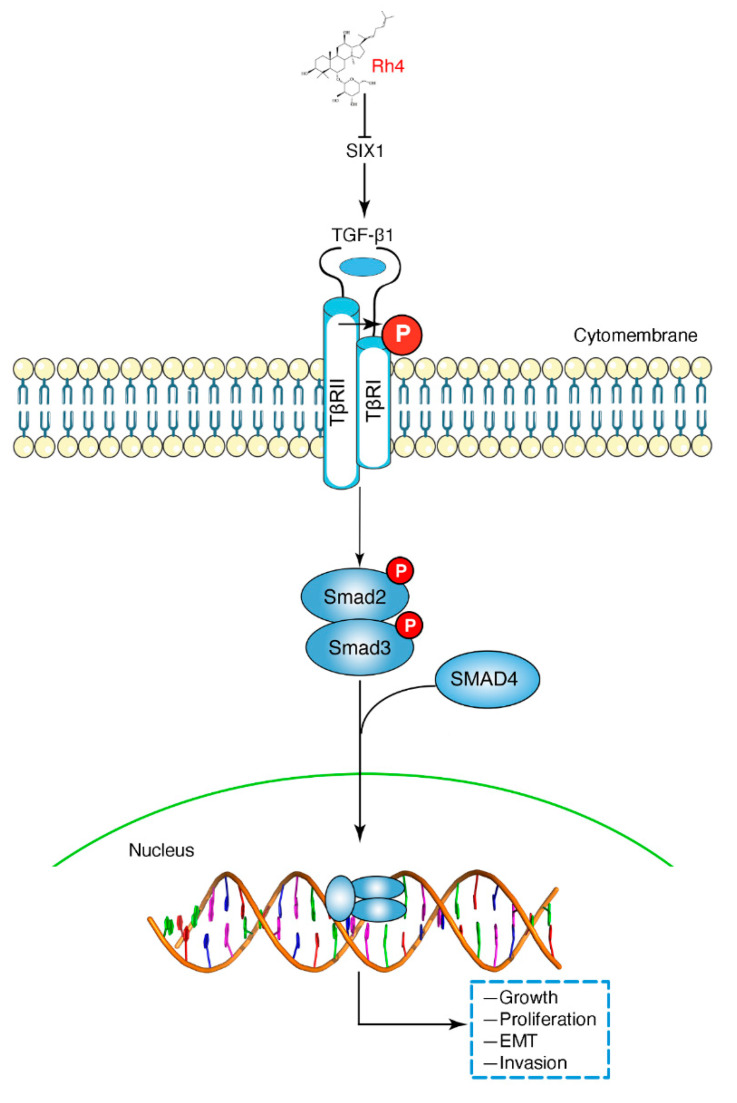
Schematic representation of the proposed mechanism of Rh4 in GC cells. Rh4 targets SIX1 to inhibit TGF-β/Smad2/3 signaling, preventing the metastasis of GC.

Ginsenoside Rh4, as the natural product of ginseng, has been confirmed to have immunomodulatory, anticancer, anti-inflammatory, and antidiabetic properties [42,43,44], demonstrating its essential role in the treatment and prevention of ranges of diseases. However, the anti-GC effect of Rh4 and the underlying molecular mechanism are largely unknown. In this research, we corroborated that ginsenoside Rh4 markedly suppressed growth, proliferation, EMT, and invasion by blocking the activation of the SIX1–TGF-β/Smad2/3 signaling axis in vitro and in vivo (Figure 7), which preliminarily revealed the anti-metastasis effects of Rh4. In vitro, according to the MTT assay, colony formation assays, and AO/EB staining, Rh4 obviously decreased the survival ratios to 20% and restrained colony formation at the dose of 120 μM. In vivo, the number of tumor nodules was 51.22% and 92.68% for 50 mg/kg and 100 mg/kg Rh4 intraperitoneal injections, respectively, thus confirming the outstanding efficacy of Rh4. Simultaneously, unlike oxaliplatin, Rh4 did not cause weight loss in tumor-bearing nude mice. In addition, H&E staining showed that the treatment with Rh4 did not negatively affect the normal structure and function of heart, lung, liver, spleen, and kidney, indicating that Rh4 has little toxicity toward the organism during GC treatment. As a result, the strong anti-GC effect and low toxicity of Rh4 suggest that it can be used as an effective drug for GC treatment.

EMT is an essential procedure in tumor metastasis, which hardly occurs in normal cells. Under the influence of various factors such as the tumor microenvironment, tumor epithelial cells lose their epithelial phenotype (e.g., epithelial polarity and cell junctions), promoting a shift to a mesenchymal phenotype, which makes the cells more invasive and migratory to other cells [45]. Studies have shown that tumor metastasis is inseparable from the continuous EMT process [46], which is often associated with EMT-related protein changes. In the EMT procedure, the most important change among these biomarkers is the decrease in E-cadherin, which keeps cells tightly adherent, and the increase in N-cadherin, a characteristic protein of mesenchymal cells, as well as Vimentin, an essential structural protein of the cell, whose expression in mesenchymal cells is higher than that in epithelial cells. In addition, expression of EMT-promoting transcription factor Snail suppresses E-cadherin transcription. From our latest research, results from transwell assays and cell scratch tests demonstrated that coincubation with Rh4 significantly inhibited the migration and invasive ability of GC cells. In vivo and in vitro, it was obtained from Western blot, qRT-PCR, and IHC analysis that Rh4 decreased the level of N-cadherin while it enhanced that of E-cadherin, suggesting that the EMT of GC cells was visibly suppressed. As obtained by Western blot, qRT-PCR, and immunohistochemical analysis, Rh4 attenuated N-cadherin expression and increased E-cadherin expression, indicating that the EMT of GC cells was significantly inhibited in vivo and in vitro.

To our knowledge, TGFβ receptor type I (TβRI) and type II (TβRII) bind io TGF-β ligands, phosphorylating Smad2/3, which plays an important role in proliferation, apoptosis, and differentiation [47,48]. The TGF-β/Smad2/3 signaling pathway was suppressed by silencing Trim59 in bladder cancer [49]. Moreover, nobiletin blocked TGFβ1/Smad3 signaling to inhibit EMT in human non-small-cell lung cancer [50]. Similarly, we confirmed that TGF-β/Smad2/3 signaling was refrained with Rh4 treatment. Western blot and immunohistochemical analysis of cellular proteins and tumor tissue proteins concluded that Rh4 significantly inhibited the expression of TGF-β1, P-Smad3, and Snail and blocked the EMT process in GC cells.

SIX1 is an essential developmental transcription factor that not only has a promotive effect on embryonic muscle, but its overexpression also promotes tumorigenesis [51]. SIX1 has been reported to promote the proliferation and metastasis of a variety of cancer cells such as breast cancer, hepatocellular carcinoma, and GC cells. According to recent studies, the SIX1-induced upregulation of TGF-β1 is critical for the propagation of TGF-β/Smad2/3 signaling, induction of EMT persistence, and metastasis [19,20]. Research from Farabaugh et al. revealed that Eya2 activates the TGF-β signaling pathway by interacting with the pro-metastatic function of SIX1 to induce EMT, thereby causing cancer epithelial cells to become cancer stem cells [20]. In our study, proteomic screening proved that Rh4 targeted SIX1 via the TGF-β/Smad2/3 signaling axis to suppress EMT. Subsequently, cotreatment with disitertide and the SIX1-siRNA transfection assay confirmed this conclusion. In conclusion, our discoveries demonstrated that Rh4 suppressed the metastasis of GC via inhibiting the SIX1–TGF-β/Smad2/3 signaling axis.

## 5. Conclusions

In conclusion, our studies confirmed that Rh4 displayed significant anti-GC effects via inhibiting the TGF-β/Smad2/3 signaling pathway, and they revealed the key role of SIX1 in this process. In metastasis and invasion models, Rh4 significantly inhibited the metastatic and invasive ability of GC cells in a dose-dependent manner in vitro. In the GC tail vein injection model, Rh4 showed significant antitumor metastatic effects with few negative effects in vivo. Proteomics combined with inhibitor experiments and SIX1-siRNA transfection assay showed that Rh4 could inhibit the TGF-β/Smad2/3 signaling pathway via binding to SIX1. Our study not only offered new insights into Rh4 as a possible anticancer agent, but also highlighted SIX1 as a potential molecular target for the regulation of Rh4 and TGF-β/Smad2/3 signaling pathways.

## Figures and Tables

**Table 1:** The organ index of mice.

Organ index	Control	Normal	Normal + H-Rh4 (100 mg/kg)	L-Rh4 (50 mg/kg)	H-Rh4 (100 mg/kg)	Oxaliplatin (10 mg/kg)
Heart	0.52 ± 0.03	0.49 ± 0.02	0.50 ± 0.04	0.49 ± 0.07	0.54 ± 0.03	0.53 ± 0.05
Liver	5.46 ± 0.25	5.43 ± 0.46	5.39 ± 0.16	5.88 ± 0.62	5.22 ± 0.18	4.79 ± 0.32
Spleen	1.45 ± 0.23	1.44 ± 0.18	1.47 ± 0.15	1.50 ± 0.19 ##	1.41 ± 0.28 ##	0.39 ± 0.12 **
Lung	0.78 ± 0.16	0.72 ± 0.09	0.73 ± 0.15	0.71 ± 0.07	0.72 ± 0.13	0.71 ± 0.15
Kidney	1.44 ± 0.16	1.47 + 0.11	1.46 ± 0.09	1.49 ± 0.20	1.45 ± 0.16	1.47 ± 0.16

Statistics are exhibited as the mean ± SD (*n* = 5 in each group); ** *p* < 0.01 compared with the control group, ## *p* < 0.01 compared with the disitertide-treated group.

## Data Availability

The data presented in this study are available within the text and figures.

## Supplementary Materials

The following supporting information can be downloaded at: https://www.mdpi.com/article/10.3390/nu14081564/s1, Figure S1: Ginsenoside Rh4 inhibits GC cells growth in vitro, Figure S2: Ginsenoside Rh4 inhibits GC metastasis in vivo, and causes low toxicity and side effects.
