# Supplementary material for: Ginsenoside Rh4 Suppresses Metastasis of Gastric Cancer via SIX1-Dependent TGF-β/Smad2/3 Signaling Pathway
Source: Nutrients. 2022 Apr 9;14(8):1564. doi: 10.3390/nu14081564 (PMC9032069; doi:10.3390/nu14081564)
Supplement: Supplementary file 1 [file nutrients-14-01564-s001.zip › nutrients-1656831-supplementary.pdf]

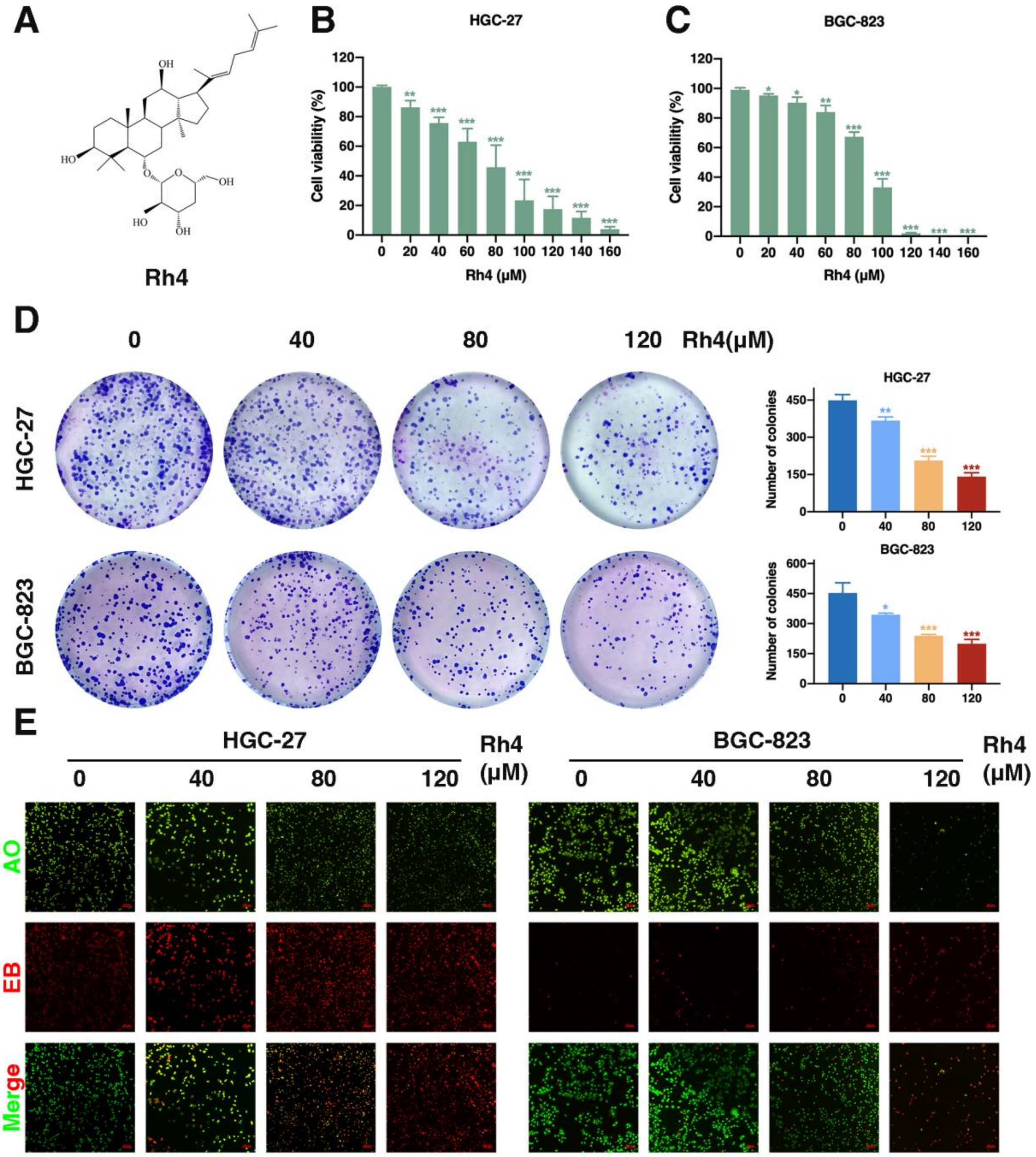

Figure S1. Ginsenoside Rh4 inhibits GC cells growth *in vitro*. (**A**) The The molecular structure of ginsenoside Rh4. (**B**, **C**) MTT assays. (**D**) Colony formation assay. (**E**) AO/EB staining. Scale bars = 100 μm. Quantification charts are listed on the right. Statistics are exhibited as the mean ± SD of triplicate independent experiments, *$p < 0.05$, **$p < 0.01$, ***$p < 0.001$.

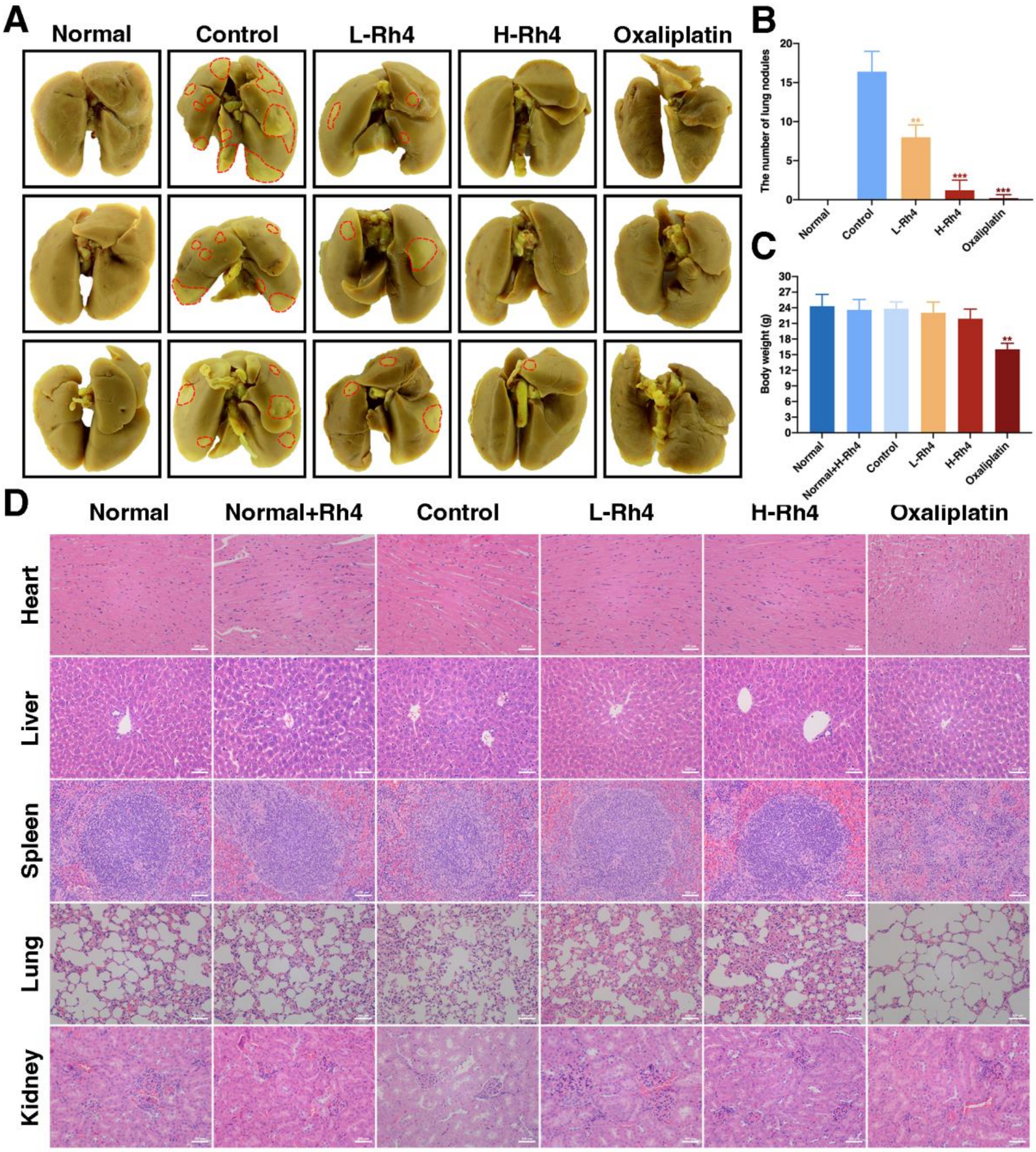

Figure S2. Ginsenoside Rh4 inhibits GC metastasis in vivo, and causes low toxicity and side effects. (**A**) Representative lung staining image of HGC-27 tail vein injection model of the normal group, control group, low-dose Rh4 group, high-dose Rh4 group, and Oxaliplatin group. (**B**) Histogram of the number of metastatic nodules in the lung. (**C**) Body weight was measured before sacrificed. (**D**) H&E staining of organ tissue. Scale bars = 200 μm. Statistics are exhibited as the mean ± SD of triplicate independent experiments, $^{**}p < 0.01$, $^{***}p < 0.001$.
